# Living-Donor Uterus Transplantation: A Clinical Review

**DOI:** 10.3390/jcm13030775

**Published:** 2024-01-29

**Authors:** Massimiliano Veroux, Paolo Scollo, Martina Maria Giambra, Giuseppe Roscitano, Alessia Giaquinta, Francesco Setacci, Pierfrancesco Veroux

**Affiliations:** 1 Vascular Surgery and Organ Transplant Unit, University Hospital of Catania, 95123 Catania, Italy; giambramartina@gmail.com (M.M.G.); giuseppe.roscitano@virgilio.it (G.R.); alessiagiaquinta@gmail.com (A.G.); francesco.setacci@unikore.it (F.S.); pveroux@unict.it (P.V.); 2 Maternal and Child Department, Obstetrics and Gynecology, Cannizzaro Hospital, 95123 Catania, Italy; paolo.scollo@unikore.it

**Keywords:** Mayer–Rokitansky–Küster–Hauser syndrome, hysterectomy, robotic, laparoscopic, live births, deceased donor

## Abstract

Uterus transplantation (UTx) is currently the only available treatment for absolute uterine factor infertility. More than 90 uterus transplantations have been performed worldwide, mostly from living donors. Living-donor (LD) UTx is a challenging surgical procedure since it poses ethical issues, and it is a high-risk and invasive surgery with higher hysterectomy-related risks compared to conventional hysterectomy. A total of 59 living-donor hysterectomies have been reported in the literature, including 35 performed with a laparotomic approach, 20 with a robotic approach and 4 with a laparoscopic approach. The mean donor age was 45.6 ± 9.1 years, and 22 were unrelated with the recipients, 34 were emotionally related (27 mothers, 5 sisters, 2 mother’s sisters). The mean recipient age was 28.8 ± 4.5 years. Mayer–Rokitansky–Küster–Hauser syndrome was the most common indication for uterus transplant. Robotic living-donor hysterectomy had the longest operative time but resulted in a lower blood loss and postoperative stay compared to laparotomic and laparoscopic approaches. Twenty-nine births from LD-UTx have been reported, four after robotic living-donor hysterectomy and twenty-five after a laparotomic procedure. UTx is now an effective treatment for women with UFI. While living-donor UTx in some cases may be considered an experimental procedure, it offers the extraordinary possibility to give women the opportunity to have a pregnancy. Many efforts should be made to reduce the potential risks for donors, including the use of mini-invasive techniques, and the efficacy of UTx in the recipients, giving the potential harm of immunosuppression in a recipient of a non-life-saving organ.

## 1. Introduction

Uterus transplantation (UTx) represents an emerging approach for women with uterine factor infertility (UFI), related either to an iatrogenic cause (e.g., hysterectomy for a benign disease, postpartum bleeding, or Ashermann syndrome) or a congenital cause (uterine agenesis in Mayer-Rokitansky-Küster-Hauser (MRKH) syndrome or partial uterine malformation) [1,2,3,4,5]. Uterus transplantation is unique in the field of solid-organ transplantation, since it is not intended to cure a chronic illness leading to death or a progressive worsening of quality of life, but it aims at restoring anatomical normalcy in women with UFI, giving them the possibility of carrying their own pregnancy and delivering their children. In this view, UTx represents an alternative treatment for UFI to adoption or gestational surrogacy [6]. Moreover, UTx is a temporary transplant, because it can be removed once the mother has delivered her child or children, and the ability to give a live birth represents the measure of the success of this transplantation, rather than its longevity [6]. 

After the first successful uterus transplantation performed in Turkey from a deceased donor [7,8], Brännström et al. [9] in Sweden reported the first successful live birth after uterus transplantation from a living donor, and the uterus transplantation has become more attractive for women with UFI, particularly those with MRKH syndrome [10]. A recent study [11] reported a web-based survey conducted among 148 MRKH patients and found that 88% of participants reported a desire for parenthood, and 61% opted for UTx as their first choice to reach this aim. Interestingly, only 13% of participants changed their mind after full information about the uterus transplantation, highlighting the great expectation for this procedure. An interesting study from Japan [12] found that 32% of female respondents may well seek to become a donor if one’s daughter suffered from UFI, while in Sweden, 80% of a population of women 30–39 years of age supported the UTx in UFI [13]. The first report of the Registry of the International Society of Uterus Transplantation [2] reported 45 UTx procedures with 19 newborns, most of which (78%) were performed from a living donor (LD), but with additional personal communications from all centers discussed at the Third International Congress of the International Society of Uterus Transplantation and the press release, a total of 91 UTx (71 LDs and 25 DDs) have been performed worldwide, resulting in 49 live births, 40 after LD UTx and 9 after DD UTx [14,15,16,17,18]. Living-donor UTx is a challenging surgical procedure since it poses ethical issues, and it is a high-risk and invasive surgery with higher hysterectomy-related risks compared to conventional hysterectomy [1,11,19]. This review explores the current status of UTX from a living donor, evaluating the potential harm and risks related to this procedure and the recent advancements in surgical technique.

## 2. The Living Donor: Is It the Right Choice?

The first living-donor uterus transplantation resulting in healthy childbirth was performed in Sweden in 2012, following several years of basic research and clinical studies [20,21]. As in every organ transplantation from a living donor, the most important aim to achieve is to preserve donor’s health: UTx is not a life-saving transplant, so only slight harm to the donor is acceptable [22]. A living donor is potentially the best resource for UTx, since it is associated with a better histocompatibility when using a direct donor [23]. However, living-donor hysterectomy is a challenging and long surgical procedure with a higher risk compared to conventional hysterectomy. Most common complications include urinary tract complications, infection, bleeding, thrombosis, and hematoma [24], bowel injuries, urinary tract infections, and iliac vessels and ureter injury [24], with more than 1 in 10 LDs requiring a surgical intervention following uterine donation due to postoperative complications [25,26]. The long surgical duration for donor surgery in LD-UTx may increase the risk of thrombo-embolic events, particularly pulmonary embolism: this life-threatening complication may be prevented but not eliminated with pre-operative and postoperative anticoagulation and early mobilization after surgery [25]. If the living donor hysterectomy is performed in a premenopausal age, there is an increased risk of early menopause [21] due to the injury to the ovarian blood flow and excision of the ovaries, which could lead in turn to long-term health risk because of the sudden cessation of ovarian-derived estradiol, which will increase the long-term risk for cardiovascular disease [21]. However, most of these complications have been prevented with improved skill and expertise in living-donor hysterectomy and with the introduction of mini-invasive techniques, such as robotic hysterectomy [27]. Living donors should be thoroughly informed about the risks and benefits of the donation, with special attention to social pressures and any possible coercion [5,28]. Moreover, it should be emphasized that the LDs revoke all parental rights to any resulting children gestated from the donated uterus, and that a future relationship with the child is by no means guaranteed [28].

On the other hand, the current principles of organ transplantation still would prefer that the organ should be collected from a deceased donor. In principle, a deceased donor should be preferred because it allows for a faster procurement time and for a greater length of vascular pedicles [19], thereby reducing one the main challenge of living-donor hysterectomy. However, uterus procurement technique from a deceased donor is not well established and given that it is not a life-saving organ, the timing of procurement may be conflicting with that of vital organs. Moreover, deceased-donor hysterectomy is not preventable, and patient selection could be more difficult. However, the procurement of a uterus from a deceased donor could be faster and simpler compared to LDs, since the ureters can be cut on both sides of the uterine vascular pedicles, preventing the extensive, meticulous, and time-consuming dissection of the ureteric tunnel in LDs [26]. Furthermore, UTx from DD may prevent the risk of physical and psychological harms to the donor and the DDs are usually younger and at premenopausal age, which could constitute an ethical problem for LDs [23]. On the other hand, cold ischemia time is increased for DD grafts compared with LD grafts, whereas LD grafts have longer mean warm ischemia time [29].

While there is a potential deceased donor availability for uterus transplantation, only 1–8.5% of all deceased donors are finally considered potentially suitable for uterus transplantation [14,30,31]. On the other hand, LDs have a careful preoperative assessment, including preoperative uterine imaging for assessing uterus vasculature, which is very difficult to plan in deceased donors [5]. This careful evaluation increases the likelihood of transplanting a uterus with a high chance of survival and high probability of pregnancy and childbirth [1,5]. Furthermore, a living-donor UTx may be planned and is associated with a shorter time on the waiting list. Since uterus is not a life-saving organ, it is the final procurement after vital organs and this would significantly increase the cold ischemia time in a deceased donor, when compared with a LD, and the real tolerance of the uterus to cold ischemia time has not been yet elucidated [19]. Finally, the microvessels located in tissues surrounding the uterus must be carefully ligated during procurement and bench surgery, to prevent post-reperfusion bleeding in the recipient. Recent studies reported an increased risk of bleeding in the recipient of a uterus from a deceased donor, mainly after reperfusion [32], although more recent studies did not confirm this assumption, showing similar rate of blood loss between UTx from LDs and DDs [23]. Deceased-donor UTxs have similar outcomes in terms of technical success, first menstruation time, and livebirth rate compared with those from LDs [29].

Due to these areas of uncertainty, LDs should be preferred in centers with a high experience with mini-invasive hysterectomy. However, the increasing number of UTxs from deceased donors performed worldwide will provide more experience and evidence about the safety and efficacy of this procedure [33]. In this view, if comparable results are achieved, LD-UTx may no longer be ethically justifiable provided the deceased donor pool can provide a sufficient supply [28].

## 3. Psychological Aspects of Uterus Transplantation

Most of UTx procedures worldwide have involved women with MRKH syndrome, which may negatively affect the psychological status of a woman, who typically is diagnosed during the sensitive adolescence period [34,35], by impairing their quality of life and sexual self-esteem [35]. This leads to a great expectation in UTx, and the birth of at least one child is the goal, although it may take several years from the UTx until this is achieved [34]. Moreover, in uterus transplantation from living donors, the recipient might experience fear for the donor health together with embarrassment, anxiety, and guilt for the involvement of a healthy donor [36,37,38]. LDs, such as mothers and sisters, may claim a right to children born from donated uteri and this may lead to internal pressure for reception of a living donor uterus transplantation [1]. On the other hand, the concerns of LDs about their hysterectomy surgery and later, about the reproductive outcome and health of the respective recipient, may also affect their psychological health and quality of life [34]. Living uterus donors may experience several mental pathological conditions, including anxiety and depression [39], but also a distortion of body image, decreased quality-of-life changes in sexual libido, and increased sexual dysfunction [36,37,38]. Moreover, the surgical scars can distort body image, and most women might feel unattractive following the hysterectomy [39], although this aspect has been improved with mini-invasive surgical techniques. However, after an UTx between related individuals, the positive effect of having a healthy child might last for many years [1], and LDs might enjoy the relationship with the born children [40]. In a recent study, Järvholm et al. [34] explored the psychological outcomes after uterus transplantation in both donor and recipient 5 years after transplantation: most recipients had a better quality of life compared to the general population but a declining satisfaction with their marital relationship. The LDs had mental components of quality of life above the predictive value of the general population with a stable marital satisfaction compared to baseline levels. However, failure to achieve a live birth may negatively affect quality of life and the grade of anxiety and depression for both donor and recipient [34]. Similar outcomes were reported even in nondirected uterus donors [41], who did not report psychological distress one year after donation, although some donors might experience an increase in depression symptoms and a decline in emotional well-being.

## 4. Surgical Issues of Uterus Transplantation

### 4.1. Living Donor

Uterus procurement from a living donor is a challenging surgical procedure requiring up to 10–13 h to be completed [42], mostly due to the difficulty in handling the complex venous system around the uterus [20,21,22,42,43]. The technique has been extensively described by Brännström et al. [20,21,42,43]. As in conventional hysterectomy, donor hysterectomy may be performed either by laparotomy or robotics. Laparoscopic and robotic surgery have been recently used in many LD procedures in other solid-organ transplants: in the setting of UTx, the laparoscopic and robot-assisted living-donor UTx operations tended to reduce blood loss compared to the open approach, with an early discharge compared with the open approach [27]. However, the operative time of robot-assisted procedure may be significantly longer and resulted in less live births compared to an open procedure [27] in which, in contrast, surgical time and warm ischemic time are considerably shorter with the additional opportunity to assess the quality of vessels through palpation, and could have, in principle, a superior ability to preoperatively assess graft quality [14]. However, an open procedure has a higher incidence of postoperative surgical complications and graft failure [27]. A total of 59 living-donor hysterectomies have been reported in the literature (Table 1), including 35 performed with a laparotomic approach, 20 with a robotic approach and 4 with a laparoscopic approach [6,15,16,20,22,44,45,46,47,48,49,50,51,52,53,54,55,56,57]. The mean donor age was 45.6 ± 9.1 years, and 22 were unrelated with the recipients, 34 were emotionally related (27 mothers, 5 sisters, 2 mother’s sisters), while in three cases, the relationship was not reported. The mean recipient age was 28.8 ± 4.5 years, and the MRKH syndrome was the most common indication for uterus transplant (52 patients), while 2 patients required UTx after hysterectomy for myomectomy and 1 patient following hysterectomy for cervical cancer [6,15,16,20,22,44,45,46,47,48,49,50,51,52,53,54,55,56,57].

With the open technique, a subumbilical midline incision is used while five working ports are used in robotics. The most sensitive part of the LD hysterectomy is to retrieve vascular pedicles, particularly for uterine veins, of sufficient length to perform a safe vascular anastomosis; moreover, another critical point is the dissection of the ureteric tunnel when releasing the uterine vascular pedicle [20,42,43]. The surgical procedure usually begins with the transection of the round ligaments to open the vesicovaginal space. The dissection of the ureteric tunnel may be challenging, particularly in the distal part of the ureter, which overrides the uterine artery and the deep uterine veins. The distal part of the ureter is usually close to the deep uterine veins and to small vessels, which should be divided. This step may be particularly long and time-consuming, and some centers have proposed the use of the uterine branches of the utero-ovarian vein [14,20,22,42,43]. However, using the full length of the utero-ovarian vein bilaterally requires an oophorectomy, which can be considered only in women of postmenopausal age [14].

When the uterine veins are not preserved, the operative time is reduced, irrespective of the technique used, with a higher rate of postoperative complications, particularly with robot-assisted approach [27]. However, the correct venous outflow is key for the success of UTx: some authors also suggest a careful observation of the venous outflow while on the back table and after reperfusion of the uterus, reserving the need for an additional anastomosis to the external iliac vein with a nonanastomosed vein with a good outflow, thus minimizing the risk of venous congestion [27]. After completing the isolation of the ureteric tunnel, the bilateral vascular pedicles on the arterial side (the uterine artery with/without a small portion of the internal iliac artery) and the venous side (deep uterine veins and or utero/ovarian veins with a segment of the internal iliac vein) are then dissected [20,21,22,42,43]. Some authors suggest that if preoperative imaging of the uterus vasculature indicates a short distance between the first major branch and the uterine artery, it may be preferable not to try to preserve the iliac branches on that side [22]. Finally, before procurement, the utero-ovarian ligaments and the sacro-uterine ligaments are divided, and the vagina is transected 2 cm below the cervix. The procedure is completed with vascular clamping and transection and immediate flushing and cooling of the uterus on the back table. While on the back table, all potential outflows should be evaluated, and the most dominant vein could be used for anastomosis, if length and quality are sufficient. A direct anastomosis to the external iliac vein with the utero-ovarian vein can be performed when deep uterine veins are not available [14]. However, many concerns may arise about the use of utero-ovarian vein and ovarian vein as drainage veins since they could not be sufficient for blood flow in the gestational uterus [27]. However, live birth is possible even without using the uterine veins as a drainage vein, suggesting that the venous flow by the gestational uterus is preserved even if only the utero-ovarian vein and/or the ovarian vein are used as drainage veins [27]. Special care should be used when using the ovarian vein in a premenopausal donor due to the potential higher risk of loss of ovary function, so that the use of ovarian veins in premenopausal women is not recommended if this results in the loss of ovaries [58]. Robotic living-donor hysterectomy had the longest operative time (11 h 45 min ± 2 h 21 min) compared to the laparoscopic approach (3 h 30 min ± 0 h 33 min) and laparotomic surgical technique (8 h 10 min ± 30 min). Blood loss was significantly lower in robotic hysterectomy (202.22 ± 469 mL) compared with a laparotomic procedure (720.31 ± 566.89 mL), and postoperative stay was lower for robotic hysterectomy compared with a laparotomic procedure (5.13 ± 2.7 vs. 7.1 ± 2.6, days, respectively) [6,15,16,20,22,44,45,46,47,48,49,50,51,52,53,54,55,56,57]. Two retrieved grafts were not transplanted because of poor venous outflow and the failure to provide adequate flow through uterine arteries during back-table preparation [22,57]. This would increase the risk of immediate transplant failure, thus highlighting the need for preoperative imaging of the donor vasculature to exclude cases at high risk for low blood flow [59]. In the uterus of living donors, a magnetic resonance angiogram (MRA) could be useful to acquire valuable details of uterine arteries. However, in 43% of cases, the uterine arteries may not be fully visualized by MRA, and this mandates the need for a computed tomography angiography [59]. However, magnetic resonance, MRA, and computed tomography angiography are equally efficient in estimating the diameter of uterine arteries [14,59]. Complication in LDs have been reported in 17% of donors, most of which related to the urinary system, mainly related to the difficult dissection of deep uterine veins close to the ureteric tunnel [21,22]. Ureteral complications include hydronephrosis, presumably due to thermal injury and a consecutive stricture of the ureter [22], but also ureteric lacerations [22,44] that were corrected during surgery, and one ureterovaginal fistula, corrected with a reimplantation of the ureter 4 months after hysterectomy [20]. Alternative strategies to reduce the incidence of such complications include using the ovarian branches of the utero-ovarian veins with an anastomosis to the external iliac veins, without the need for an oophorectomy [6], the use of ureteric stents, and the use of indocyanine green to identify ureters and vessels [21,22].

### 4.2. Recipient

Uterus transplantation is not without risk for both mother and fetus: this imposes clear information about the potential risk related to transplantation including the need for immunosuppression. Moreover, the UTx recipients should be clearly informed about the possibility of organ removal before pregnancy due to medical or surgical complications or graft rejection, and this may lead to complex emotional, ethical, and medical issues regarding the termination of a highly desired pregnancy [22]. Furthermore, the consent should describe the difference in pregnancy experience in UTx, where the iliac nerves are transected during donor surgery, and this could increase the possibility that the mother will not feel fetal movements or experience contractions and other sensations normally felt when pregnant [22].

The recipient surgery is similar regardless of the type of donor. The duration of the open procedure is reported as 2–6 h in 73% of cases [20], while the first robotic UTx has been recently performed [14]. The surgery starts with the removal of the rudimentary uterus, with the clearance of the vaginal vault from the bladder. The external iliac vessels are dissected. Uterine veins and/or utero-ovarian veins are anastomosed end-to-side with the external iliac vein on both sides, while the uterine artery with the iliac patch is anastomosed end-to-side to the external iliac artery on both sides. The vault is opened and a vaginal–vaginal anastomosis is accomplished. Fixation sutures connect the round and the sacro-uterine ligaments. An echo-color doppler of the uterine vessels is finally performed before wound closure [33]. The most common complication in uterus transplantation is the graft failure: a recent review [17] reported an overall graft failure of 19.8% (19/96), 16.9% (12/71) from living donors and 28% (7/25) from deceased donors; however, some of the reports came from unpublished data, and it is likely that the rate of graft loss between LDs and DDs could be comparable [17]. Moreover, intravascular thrombosis was a more common cause of graft failure in UTx procedures from living donors than those from deceased donors [17].

Among the 59 LD UTx reported in the literature, a total of 11 grafts were lost (18.6%), leading to an overall surgical success of UTx of 71.4%: surgical success was achieved in 75% of laparotomic LD-UTx, which was lower than laparoscopic LD-UTx (100%) and robotic LD-UTx (90%). The main causes for graft loss included vascular thrombosis (eight grafts), recurrent infections (one graft), venous outflow obstruction (two grafts), and poor reperfusion after vascular declamping (one graft) [6,17,20,22,42,43,44,48,49]. The mean time from transplant to graft failure was 50.3 ± 72 days [17]. Although an overall higher technical success was reported with robotic hysterectomy, it should be highlighted that this may be misleading because all programs of living-donor hysterectomy started with the laparotomic approach and moved to the robotic approach after developing a sufficient comfort.

Uterus living donors are usually in postmenopausal age, and several studies have demonstrated that, after menopause, the size of the uterus decreases, and atherosclerosis might progress, thus reducing the uterine vasculature [1] and increasing the risk of poor graft reperfusion and thrombosis.

A late complication of UTx, which typically occurs several months after UTx, is vaginal stricture over the suture line, which may affect up to 72% of recipients, with half of them treated by nonsurgical dilatation and the rest by surgery [21]. Histocompatibility, as in other solid organ transplantation, may have a role in reduced graft function: however, most LD-UTxs are performed using intrafamilial LDs, and this significantly reduces the risk of acute rejection [5]. Almost all UTX recipients receive an induction therapy with basiliximab or thymoglobulin + steroids together with a triple-drug maintenance therapy with tacrolimus, mycophenolate, and steroids. Mycofenolate is usually discontinued at the time of the first embryo transfer due to its teratogenicity and replaced with azatiophrine [2,60]. At our center, UTx recipients usually receive an induction with thymoglobulin + steroids and a maintenance therapy with tacrolimus, mycophenolate, and steroids. Mycofenolate is usually replaced with azathioprine 6–8 months after transplantation, when the first embryo transfer can be planned [33]. However, Jones et al. [61] suggested that although azathioprine was safe to take during pregnancy with no increased risk of congenital abnormality, an association with preterm delivery and low birth weight could not be excluded.

Uterus transplant may be monitored by the mean of cervical biopsies, and up to 23% of recipients may experience an acute rejection episode, usually mild or moderate, treated with a steroid bolus [2]. The chronic exposure to immunosuppression of uterus transplant recipients is associated with a decrease in glomerular filtration rate (GFR) early post-transplant, which could persist even in the early postpartum period [62,63]. Johannesson et al. [62] evaluated the decline in renal function in 22 UTx recipients: the mean GFR at last follow-up (92.1 mL/min per 1.73 m^2^) was significantly lower than the pretransplant eGFR (106.4 mL/min per 1.73 m^2^, *p* = 0.001); interestingly, although a rebound of the GFR was observed early after hysterectomy and immunosuppression withdrawal, at the 3-year follow-up, UTx recipients displayed a persistent reduction in eGFR of 10.2 mL/min per 1.73 m^2^ compared with pretransplant levels, and the risk of a lower GFR was particularly higher in women experiencing acute kidney injury or preeclampsia during pregnancy [62]. Uterus transplantation is a temporary transplant, and graft hysterectomy (GH) is planned either at the time of delivery or at a later date [50,62]. While GH is usually performed with a traditional open approach, Finotti et al. [64] recently presented the first two cases of robotic GH in UTx. The advantages of a robotic technique are a better control of hemostasis, better operative field vision particularly useful in the presence of adhesions, and superior intraoperative maneuverability, together with less postoperative pain and a shorter length of stay [64]. Brucker et al. [65] reported the first successful laparoscopic GH three months after delivery in a young LD-UTx recipient who had developed an impaired renal function with bilateral hydronephrosis during pregnancy.

## 5. Live Birth

According to the recent literature reported in Table 1, 29 live births from LD-UTx have been reported so far, 4 after robotic living-donor hysterectomy and 25 after a laparotomic procedure [6,20,22,45,46,47,48,49,50,51,52,53,54,57]. Almost all deliveries were by Caesarean section, and all occurred with a median gestational age at birth between 35 completed weeks (range: 31–38 weeks) [2] and 36 weeks and 6 days (range: 30.1 to 38.0 weeks) [29]. Among the 18 live births reported by Johannesson et al. [66], planned term deliveries occurred in 44% (8/18) of live births, while unplanned deliveries occurred more frequently in women with spontaneous preterm labor, severe rejection, subchorionic hematoma, and placenta previa.

Almost half of UTx neonates may require at least 1 day in neonatal intensive care [29], mainly due to respiratory distress syndrome [45]. Although children born after UTx are in utero exposed to immunosuppression, most of the infants had a neonatal course that reflected the gestational age at delivery, and no baby was born with an identified malformation or organ dysfunction [67]. At the 2-year follow-up, all children’s growth and physical, neurological, and cognitive developments were age-appropriate within the first 2 years of life [68].

## 6. Future Perspectives

Although increasing numbers of UTx have been performed during the last decade, many aspects concerning ethical consideration, surgical issues, and postoperative management have not been completely elucidated and need to be standardized.

It is likely that UTx could be offered to a growing number of women with UFI, not only because of MRKH syndrome but also for a hysterectomy for benign disease. However, many ethical concerns still persist. Although there is a general agreement that UTx could be beneficial for women with UFI [11,12,13], in the USA, only 45% of surveyed reproductive endocrinologists and gynecologists felt that UTx could be a safe alternative for UFI patients, due to the potential high risk of medical and surgical complications [69]. Another important issue is the costs of UTx. In many countries, UTx is not covered by the public healthcare system: a recent study from Denmark [11] evaluated the estimated total costs for LD UTx at EUR 93850, including preoperative investigations, transplantation surgeries, 2-year follow-up with immunosuppressive therapy, and hysterectomy, and the authors concluded that the potential benefits of UTx do not justify the associated risks and costs of the procedure [11]. Moreover, some authors have argued that the existential suffering addressed by UTx does not possess a sufficiently strong normative value to legitimize such a high-risk and expensive procedure [70]. In this view, UTx may represent an inappropriate use of limited healthcare resources towards life-threatening conditions that should be prioritized over non-life-saving conditions such as UTx [71]. However, in some European regions, including Italy, UTx is now covered by the national health system under a restricted clinical protocol, and this would increase the likelihood for women with UFI to be scheduled for UTx.

As UTx becomes a more routine procedure, more people will be evaluated for becoming potential recipients of UTx. Usually, UTx has been proposed only to genetically XX females, but probably in the future, it may be expanded to genetically XY people, including transgender male-to-female people [72]. In this setting, UTx could constitute an opportunity for some transwomen to contribute to the success of gender transition, although this may face legal, religious, and moral obstacles, including the appropriate designation of parenthood [73].

One of the major limitations for the widespread adoption of UTx as a treatment for UFI is the donor availability. A potential recipient rarely has a suitable LD, and very few females have uteri suitable for donation [21]. One possible solution is the nondirect LD uterus donation which has been extensively practiced with success [52,54], especially with the use of robotic hysterectomy. However, special care should be devoted to donors <40 years, where an extensive psychological assessment is mandatory to be certain that they would not later regret their permanent loss of childbearing capacity [21]. Another option to increase the donor pool would be to reuse a transplanted uterus after planned hysterectomy in a first recipients after a live birth [21], since the uterus could be easily procured with long vascular pedicles, but the chronic rejection and the progressive aging of the uterus could significantly affect the outcome of a retransplanted uterus. Another potential way to increase the donor pool is to accept older donors, as already done in other solid organ living transplantations: with a careful predonation imaging evaluation of uterine arteries’ calibers [59], LD UTx is potentially feasible even from donors >60 years [46]. A futuristic opportunity is the bioengineered uterus, which could overcome the shortage of suitable uterus donor by using a scaffold, which is colonized by the patient’s own cells to generate patient-specific uterine material [21], as has already been reported for liver 3D bioprinting [74].

## 7. Conclusions

Thanks to a fully translational approach, including animal studies and clinical trials, UTx is now an effective treatment for women with UFI. Living-donor UTx could be now considered an emerging surgical procedure, since it offers the extraordinary possibility to give women the opportunity to have a pregnancy. Many efforts should be made to reduce the potential risks for donors, including the use of mini-invasive techniques, and the efficacy of UTx in the recipients, given the potential harm of immunosuppression in a recipient of a non-life-saving organ. Moreover, some ethical concerns about the feasibility, acceptability, and above all, the sustainability of UTx transplantation should be evaluated on a cost-to-benefit ratio. However, as experience increases, the safety and efficacy of the LD, recipient, and child will improve, and costs will probably decrease, and this could be a step forward to pave the way for UTx to become the preferred infertility treatment for women with UFI.

## Figures and Tables

**Table 1:** Living-donor uterus transplantation: analysis of the current literature.

Reference	Year	Number of Cases	Donor Age (Years)	Recipient Age (Years)	Relationship	Indication for Transplant	Surgical Technique	Donor’s Operative Time (h)	Blood Loss (mL)	Postoperative Discharge (Days)	Graft Failure	Success Rate	Live Birth
Fageeh et al. [44]	2000	1	46	26	Unrelated	Hysterectomy for postpartum bleeding	Laparotomy	NA	NA	NA	Yes (graft failure for vascular thrombosis)	0%	0
Brännström et al. [20]	2014	9	52, 54, 58, 61, 50, 53, 50, 37, 52 (total 53.0 ± 7.0)	33, 38, 28, 27, 35, 27, 28, 33, 35 (total 31.5 ± 3.9)	Mother (5), mother-in-law (1), mother’s sister (1), sister (1), unrelated (1)	MRKH (8), Hysterectomy for cervical cancer (1)	Laparotomy	12.1 (mean)	922 ± 772	6	2/9 (1 graft failure for graft thrombosis, 1 for recurrent infections)	75%	9
Brännström et al. [45,46]	2020	8	49, 62, 55, 48, 45, 57, 37, 46 (total 49.8 ± 7.8)	22, 32, 33, 29, 24, 30, 31, 23 (total 28 ± 4.3)	Mother (6), sister (1), unrelated (1)	MRKH (8)	Robotic	11.5 ± 0.9	500 ± 221 (mean)	5 (7 NR)	2/8 (2 hysterectomy for graft necrosis)	75%	1
Wei et al. [47]	2017	1	42	22	Mother	MRKH	Robotic	6	100	5	No	100%	1
Puntambekar S et al. [48,49]	2018	4	45, 42, 48, 47 (total 45.5 ± 2.6)	26, 21, 24, 30 Total (25.2 ± 3.7)	Mother (4)	MRKH (4)	Laparoscopic	3.5 ± 1.1	100	7 (2) + 6(2)	No	100%	NR
Testa et al. [6,50,51]	2020	13	42, 56, 45, 34, 36, 39, 35, 48, 32, 33, 39, 32, 43 (total 39.5 ± 7.1)	31, 33, 34, 29, 27, 24, 22, 29, 20, 23, 30, 21, 31 (total 27.3 ± 4.7)	Unrelated (12), related (1)	MRKH (11), myomectomy (2)	Laparotomy	6.5 ± 0.7	873 ± 441 (mean)	5.2 (mean)	5/13 (2 outflow obstruction, 1 arterial thrombosis, 1 poor reperfusion, 1 graft ischemia)	62%	11
Testa et al. [6,50,51,52]	2021	8	30, 30, 37, 32, 38 (total 33.4 ± 3.8)	30, 34, 33, 34, 29 (total 32 ± 2.3)	Unrelated (5)	MRKH (5)	Robotic	10.5 ± 1.2	114 ± 66.9	4 (mean)	No	100%	1
Akouri et al. [53]	2020	1	50	24	Mother	MRKH	Laparotomy	10	900	7	No	100%	1
Fronek et al. [54]	2021	6 (1 not transplanted)	53, 58, 47, 49, 48 (total 51 ± 5)	30, 26, 23, 25, 26 (total 28 ± 3)	Mother (4), mother’s sister (1)	MRKH (5)	Laparotomy	6 ± 0.5	500 ± 440 (mean)	8 (mean)	1/5 (1 venous thrombosis)	80%	2
Brucker et al. [22]	2020	5 (1 not transplanted)	46, 46, 56, 32 (total 45 ± 9)	23, 23, 32, 35 (total 28 ± 6)	Mother (3), sister (1)	MRKH (4)	Laparotomy	10 ± 1	100 (mean)	12.7 ± 1.5	No	100%	2
Viera et al. [55]	2021	1	50	33	Unrelated	MRKH	Robotic	8	NA	2	No	100%	NR
Carmona et al. [56]	2021	1	NA	31	Sister	MRKH	Robotic	10	NA	4	No	100%	NR
Ayoubi et al. [57]	2022	1	57	34	Mother	MRKH	Robotic	13	150	11	No	100%	1
Deans et al. [15]	2023	1	47	25	Unrelated	MRKH	Laparotomy	10	750	8	No	100%	NR
Jones et al. [16]	2023	1	40	34	Sister	MRKH	Laparotomy	8	900	5	No	100%	NR

Legend: MRKH: Mayer–Rokitanski–Küster–Hauser syndrome; NA: not available; NR, not reported.

## Data Availability

It is possible for deidentified data to be made available upon reasonable request.
